# Associations between food insecurity and diabetes risk factors in US adolescents in the National Health and Nutrition Examination Survey (NHANES) 2007–2016

**DOI:** 10.1017/S1368980024000284

**Published:** 2024-02-12

**Authors:** Aarohee P Fulay, Joyce M Lee, Ana Baylin, Julia A Wolfson, Cindy W Leung

**Affiliations:** 1 Department of Epidemiology, University of Pittsburgh Graduate School of Public Health, 130 N. Bellefield Ave., Pittsburgh, PA 15213, USA; 2 Susan B. Meister Child Health Evaluation and Research Center, Division of Pediatric Endocrinology, University of Michigan Medical School, Ann Arbor, MI, USA; 3 Department of Nutritional Sciences, University of Michigan School of Public Health, Ann Arbor, MI, USA; 4 Department of Epidemiology, University of Michigan School of Public Health, Ann Arbor, MI, USA; 5 Departments of International Health and Health Policy and Management, Johns Hopkins Bloomberg School of Public Health, Baltimore, MD, USA; 6 Department of Nutrition, Harvard T.H. Chan School of Public Health, Boston, MA, USA

**Keywords:** Food insecurity, Diabetes, Adolescents, USA

## Abstract

**Objective:**

To evaluate the associations between household food insecurity and diabetes risk factors among lower-income US adolescents.

**Design:**

Cross-sectional analysis. Household food security status was measured using the 18-item Food Security Survey Module. Simple and multivariable linear and logistic regressions were used to assess the association between food security status and fasting plasma glucose (FPG), oral glucose tolerance test (OGTT), HbA1C and homoeostatic model assessment – insulin resistance (HOMA-IR). The analyses were adjusted for household and adolescent demographic and health characteristics.

**Setting:**

USA.

**Participants:**

3412 US adolescents aged 12–19 years with household incomes ≤300 % of the federal poverty line from the National Health and Nutrition Examination Survey cycles 2007–2016.

**Results:**

The weighted prevalence of marginal food security was 15·4 % and of food insecurity was 32·9 %. After multivariate adjustment, adolescents with food insecurity had a 0·04 % higher HbA1C (95 % CI 0·00, 0·09, *P*-value = 0·04) than adolescents with food security. There was also a significant overall trend between severity of food insecurity and higher HbA1C (*P*_trend_ = 0·045). There were no significant mean differences in adolescents’ FPG, OGTT or HOMA-IR by household food security.

**Conclusions:**

Food insecurity was associated with slightly higher HbA1c in a 10-year sample of lower-income US adolescents aged 12–19 years; however, other associations with diabetes risk factors were not significant. Overall, this suggests slight evidence for an association between food insecurity and diabetes risk in US adolescents. Further investigation is warranted to examine this association over time.

In the USA, approximately one in twelve individuals is affected by type 2 diabetes^(1)^, a chronic disease characterised by insulin resistance and high blood glucose levels^(2)^. In the long term, type 2 diabetes can cause complications such as retinopathy, neuropathy, kidney damage^(3)^ and premature death^(4)^. In 2017, the USA spent approximately $327 billion on healthcare costs related to diabetes^(5)^ and future costs may increase^(6)^. In particular, the increasing incidence of type 2 diabetes in adolescents^(7)^ is a concerning trend. Approximately one in five US adolescents aged 12–18 years is affected by prediabetes^(8)^ and the age of onset for type 2 diabetes has decreased over time^(9)^. Earlier age of onset has been associated with more rapid and severe disease progression^(10)^ including earlier complications^(11)^ and a reduction in life expectancy^(12)^. Thus, due to the prevalence and severity of this disease, type 2 diabetes in US adolescents is a critical public health issue.

Food insecurity, an important social determinant of health, has been associated with diabetes risk factors in US adults^(13)^. Food insecurity is defined by the United States Department of Agriculture (USDA) as a ‘household-level economic and social condition of limited or uncertain access to adequate food^(14)^’ and affects approximately one in ten US households^(15)^. In US adults, food insecurity is associated with poor dietary quality^(16)^ which is a known risk factor for type 2 diabetes^(2)^. Seligman *et al.* found that food insecurity was associated with 2·1 higher odds of diabetes in lower-income US adults^(13)^. In younger adults (aged 20–39 years), Lee *et al.* found that food insecurity was associated with a 1·36 higher odds of prediabetes or type 2 diabetes^(17)^.

Food insecurity affects one in eight households with children and is more prevalent than in households without children^(18)^. However, the current evidence base for the association between food insecurity and diabetes risk in children/adolescents is limited. Presently, one research study using data from Minnesota students found that food insecurity was associated with higher self-reported prediabetes risk for Non-Hispanic White, Hispanic/Latino and Non-Hispanic Black youth; however, this information cannot be extrapolated to the national level^(19)^. Additionally, Lee *et al.* found that food insecurity was associated with 1·94 higher prediabetes risk (as measured by HbA1C) in a national sample of US adolescents; however, this study did not examine if this association extended to oral glucose tolerance test (OGTT), an alternative test of glycaemia, or the homoeostatic model assessment – insulin resistance (HOMA-IR)^(20)^. Thus, this sparse evidence should be confirmed with data that use additional measures of diabetes risk factors at the national level.

The purpose of this study is to investigate the association between household food insecurity and multiple type 2 diabetes risk factors among US adolescents aged 12–19 years with household incomes ≤300 % of the federal poverty line using data from the 2007–2016 National Health and Nutrition Examination Survey (NHANES). This study would be among the first to comprehensively examine multiple diabetes risk factors (including OGTT and HOMA-IR) by adolescents’ household food security status using a large, national sample. Based on existing research, it was hypothesised that food insecurity would be associated with higher levels of all type 2 diabetes risk factors.

## Methods

### Data source

NHANES is a cross-sectional survey, which collects health and nutrition data on the non-institutionalised US population. NHANES selects participants using a complex multistage probability sampling design. From 1999 onwards, demographics, dietary, examination, laboratory and questionnaire data have been collected continuously^(21)^. Similar to a previous study^(22)^, data from cycles 2007–2008, 2009–2010, 2011–2012, 2013–2014 and 2015–2016 were pooled to ensure an appropriate sample size.

### Participants

The participants in the sample are 3412 lower-income adolescents aged 12–19 years with household incomes ≤300 % federal poverty level. The income restriction was applied to reduce the potential for confounding by income, as done in previous studies^(13,23)^. Participants were included if they had complete information on the exposure, covariates and at least one diabetes risk factor. For outcome variables that required fasting, individuals were included in the sample if they reported fasting for 9–24 h.

### Exposure

The exposure variable is household food insecurity, measured by the USDA Household Food Security Survey Module^(24)^ – a broadly used and previously validated instrument^(25)^. This module includes eighteen questions about the food security status of a household with children over the time period of the past year^(24)^ and is completed by an adult member of the household^(21)^. A score of 0–18 is created from the sum of all affirmative responses, with a higher score denoting greater food insecurity. Zero affirmative responses indicate full food security, 1–2 indicates marginal food security, 3–7 indicates low food security and 8+ indicates very low food security. For this analysis, ‘low food security’ and ‘very low food security’ were grouped into the broader category of ‘food insecurity’ per USDA guidelines^(24)^.

### Outcomes

The outcomes of interest are fasting plasma glucose (FPG), HbA1C, OGTT and HOMA-IR. For NHANES participants, all of these measures and/or their individual components are obtained at the mobile examination centre via blood samples^(21)^. FPG, HbA1C and OGTT are directly measured in NHANES^(21)^. FPG values indicate current FPG levels. HbA1C is a non-fasting measure of the percentage of glycosylated Hb in the blood and represents average plasma glucose levels over the past 3 months^(26)^. OGTT is a measure of glucose tolerance that compares a baseline FPG measurement to plasma glucose levels 2 h after the consumption of 75 g of pure glucose^(27)^. HOMA-IR is from other measures^(21)^ taken in the NHANES mobile examination centre. It is a measure of insulin resistance that is calculated from FPG and fasting insulin using the following formula: ((fasting plasma glucose in mg/dL)*(fasting insulin in uU/mL))/405 ^(28)^.

Binary versions of the outcome variables were constructed based on established cut-offs associated with higher diabetes risk^(29)^. For FPG, values less than 100 mg/dL were classified as normal FPG and values of 100 mg/dL or higher were classified as high FPG. For HbA1C, values below 5·7 % were classified into normal HbA1C and values at 5·7 % or higher were classified as high HbA1C. For OGTT, 2-h glucose values below 140 mg/dL were classified as normal OGTT and values 140 mg/dL or higher were classified as high OGTT. Because no clinical cut-off for HOMA-IR exists for US adolescents, values were considered high if they were above the 75th percentile, a categorisation previously used by Lee *et al.* ^(30)^. The unweighted number of individuals in each of the samples who had measures of each of the diabetes risk factors was: 3412 for HbA1C, 1507 for FPG, 1457 for HOMA-IR and 1323 for OGTT.

### Covariates

Covariates included age (in years), sex (male/female), race/ethnicity (non-Hispanic White, non-Hispanic Black, Mexican American, Other Hispanic ethnicity and other race/ethnicity), sedentary activity, vigorous recreational activity, moderate recreational activity, household income-to-poverty ratio, household respondent education level and household respondent marital status. Sedentary activity, classified as ‘sitting at school, at home, getting to and from places or with friends including time spent sitting at a desk, traveling in a car or bus, reading, playing cards, watching television or using a computer^(21)^’, was recoded to a binary variable such that someone who engaged in more than 360 min of sedentary activity per day was considered to engage in ‘high sedentary activity’ and someone who engaged in 360 min or less of sedentary activity per day was considered to engage in ‘low sedentary activity’. Vigorous and moderate recreational activity were recoded to binary yes/no variables: if someone responded affirmatively to the question: ‘In a typical week {do you/does SP} do any vigorous-intensity sports, fitness, or recreational activities that cause large increases in breathing or heart rate like running or basketball for at least 10 minutes continuously?’, they were considered to engage in vigorous recreational activity^(21)^. Similarly, if they responded affirmatively to the question: ‘In a typical week {do you/does SP} do any moderate-intensity sports, fitness, or recreational activities that cause a small increase in breathing or heart rate such as brisk walking, bicycling, swimming, or volleyball for at least 10 minutes continuously?,’ then they were considered to engage in moderate recreational activity^(21)^. Household income-to-poverty ratio is a variable provided by NHANES, which is calculated by dividing household income by the federal poverty line from the Department of Health and Human Services^(21)^. Household respondent education level was recoded into ‘≥ high school graduate’ or ‘< high school graduate’. Household respondent marital status was recoded to a binary yes/no variable in which ‘married/partnered’ was coded as ‘yes’ and ‘single/divorced/widowed’ was coded as ‘no’.

### Statistical analysis

For descriptive statistics, weighted means and percentages were calculated. Simple linear regressions were used to assess differences in continuous variables by food security status and Rao–Scott Chi-squared tests were used to assess differences in categorical variables by food security status. To examine the associations between household food security and diabetes risk factors, multivariable linear regression was used for continuous diabetes risk factors and multivariable logistic regression for clinical cut-points, adjusting for all study covariates. To generate a p-for-trend value, the analysis was re-run with food insecurity treated as a continuous variable rather than categorical. NHANES survey weights were utilised to create national estimates. According to NHANES, the survey weights account for: ‘the differential probabilities of selection for the sampling domains, survey nonresponse, and differences between the final sample distribution and the target population distribution^(21)^’. Complex survey weights were recalculated according to the NHANES protocol^(21)^ for pooling multiple survey cycles together to reflect the probability of being sampled in the 10-year study period. Survey weights were applied to all analyses specific to the end-point of interest, for example, mobile examination centre weights for HbA1C, fasting weights for FPG and HOMA-IR, and OGTT weights for OGTT. All analyses used survey procedures in SAS 9.4 (SAS Institute) that accounted for the complex survey design including strata, clustering and weights. Additionally, the linear regression analyses utilised robust standard errors that accounted for minor non-conformities from the traditional linear regression assumptions. Furthermore, we checked for influential outliers.

## Results

In the sample, the weighted prevalence of marginal food security was 15·4 % and the weighted prevalence of food insecurity was 32·9 %. Adolescents with food insecurity were more likely to be non-Hispanic Black, Mexican American or Other Hispanic ethnicity (*P* < 0·0001) and less likely to engage in moderate recreational activity (*P* = 0·03) (Table 1). Adolescents with food insecurity were also more likely to have a household respondent with lower educational attainment (*P* < 0·0001), who was not married/partnered (*P* < 0·0001) and with lower income (*P* < 0·0001).

**Table 1:** Associations between household food insecurity and socio-demographic variables in a lower-income (300 % federal poverty line and below) sample of adolescents aged 12–19 years in NHANES cycles 2007–2016*

	Overall (*n* 3412)		Full food security† (*n* 1550)		Marginal food security (*n* 585)		Food insecurity (*n* 1277)		
Characteristic	Mean	s e	Mean	s e	Mean	s e	Mean	s e	*P* ‡
Age (years)	15·3	0·05	15·3	0·07	15·4	0·1	15·2	0·08	0·39
HH income to poverty ratio, mean (s e)	1·43	0·03	1·67	0·03	1·23	0·05	1·15	0·04	**<.0001** §
	%	*n*	%	*n*	%	*n*	%	*n*	
Female (%)	50·3	1678	51	762	52·6	311	48·1	605	0·33
Race/ethnicity (%)									**<.0001** §
Non-Hispanic White	46·4	797	54·3	419	39·7	113	37·3	265	
Non-Hispanic Black	17·0	876	14	366	21·5	173	19·7	337	
Mexican American	19·6	952	16	391	21	156	24·6	405	
Other Hispanic ethnicity	9·3	456	7·8	197	11·4	89	10·6	170	
Other race/ethnicity	7·6	331	7·9	177	6·5	54	7·7	100	
Vigorous recreational activity in typical week (%)	57·4	1950	59·2	916	52·1	307	57	727	0·11
Moderate recreational activity in typical week (%)	52·5	1652	55·6	773	49·3	277	49·3	602	**0·03** §
Low sedentary activity (%)	28·5	1028	27·7	452	29·5	185	29·4	391	0·69
HH respondent education ≥ high school graduate (%)	69·8	2195	74·7	1066	67·1	369	63·3	760	**<.0001** §
HH respondent married/partnered (%)	62·9	2061	69·2	1005	57·6	336	55·4	720	**<.0001** §

NHANES, National Health and Nutrition Examination Survey; HH, household.

* All analyses were conducted using survey procedures that account for the complex survey design including the strata, clusters and weights.

† According to the United States Department of Agriculture (USDA), full food security is defined as ‘no reported indications of food-access problems or limitations’. Marginal food security is defined as ‘one or two reported indications—typically of anxiety over food sufficiency or shortage of food in the house. Little or no indication of changes in diets or food intake’. Food insecurity is defined as ‘a household-level economic and social condition of limited or uncertain access to adequate food’. These definitions are from the USDA Economic Research Service (ERS): https://www.ers.usda.gov/topics/food-nutrition-assistance/food-security-in-the-us/definitions-of-food-security.aspx.

‡ Rao–Scott Chi-squared tests were used for categorical variables and simple linear regressions were used for continuous variables.

§ Statistically significant estimates at alpha = 0·05 are bolded.

In examining the multivariate-adjusted associations between household food security and continuous diabetes risk factors, adolescents with food insecurity had 0·04 % higher HbA1c (95 % CI 0·00 %, 0·09 %, *P* = 0·04) compared to adolescents with full food security (Table 2). There was a significant trend between greater severity of food insecurity and higher HbA1C (*P*_trend_ = 0·045) (Table 2). The associations between household food security and continuous FPG, OGTT and HOMA-IR were not significant.

**Table 2:** Linear regressions between food insecurity and diabetes risk factors in a lower-income (300 % federal poverty line and below) sample of adolescents aged 12–19 years (*n* 3412) in NHANES cycles 2007–2016*

	Model 1†				Model 2‡			
	Estimate	95 % CI LL	95 % CI UL	*P*	Estimate	95 % CI LL	95 % CI UL	*P*
**HbA1C (%) (** ***n*** **3412)**								
Full food security§	Ref.				Ref.			
Marginal food security	0·02	−0·05	0·08	0·65	0·00	−0·07	0·07	0·99
Food insecurity	0·06	0·02	0·10	**0·007** ||	0·04	0·00	0·09	**0·041** ||
* P*-for-trend				**0·007** ||				**0·045** ||
**Fasting plasma glucose (mg/dL) (** ***n*** **1507)**								
Full food security	Ref.				Ref.			
Marginal food security	0·31	−2·56	3·17	0·83	0·16	−2·68	3·00	0·91
Food insecurity	0·33	−1·11	1·77	0·65	0·27	−1·11	1·65	0·70
* P*-for-trend				0·64				0·69
**HOMA-IR (** ***n*** **1457)**								
Full food security	Ref.				Ref.			
Marginal food security	0·08	−0·35	0·52	0·70	−0·13	−0·56	0·29	0·53
Food insecurity	0·23	−0·13	0·60	0·21	0·00	−0·40	0·40	0·99
* P*-for-trend				0·22				0·97
**OGTT (mg/dL) (** ***n*** **1323)**								
Full food security	Ref.				Ref.			
Marginal food security	−1·04	−5·68	3·60	0·66	−1·66	−6·38	3·06	0·49
Food insecurity	2·87	−1·10	6·83	0·15	3·10	−1·15	7·35	0·15
* P*-for-trend				0·19				0·18

NHANES, National Health and Nutrition Examination Survey; LL, lower limit; UL, upper limit; mg/dL, milligram/decilitre; HOMA-IR, homoeostatic model assessment – insulin resistance; OGTT, oral glucose tolerance test.

* All analyses were conducted using survey procedures that account for the complex survey design including the strata, clusters and weights.

† Model 1 is age- and sex-adjusted.

‡ Model 2 is adjusted for adolescent age, sex, race/ethnicity, vigorous recreational activity, moderate recreational activity, sedentary time; household respondent education, marital status and income (linear and quadratic term).

§ According to the United States Department of Agriculture (USDA), full food security is defined as ‘no reported indications of food-access problems or limitations’. Marginal food security is defined as ‘one or two reported indications—typically of anxiety over food sufficiency or shortage of food in the house. Little or no indication of changes in diets or food intake’. Food insecurity is defined as ‘a household-level economic and social condition of limited or uncertain access to adequate food’. These definitions are from the USDA Economic Research Service (ERS): https://www.ers.usda.gov/topics/food-nutrition-assistance/food-security-in-the-us/definitions-of-food-security.aspx.

|| Statistically significant estimates at alpha = 0·05 are bolded.

Table 3 shows the associations between household food security and binary diabetes risk factors. There was a marginally significant association between food insecurity and clinically elevated HbA1C (> = 5·7 %) (OR 1·61, 95 % CI 1·10, 2·36, *P* = 0·06), and the overall trend for greater severity of food insecurity and clinically elevated HbA1C was statistically significant (*P* = 0·01) for Model 1, adjusting for age and sex. However, the association was attenuated in the multivariable-adjusted model (OR 1·37, 95 % CI 0·95, 1·98, *P* = 0·12, *P*_trend_ = 0·09). Similar to Table 2, the associations between household food security and clinical cut-points for FPG, OGTT and HOMA-IR were not significant.

**Table 3:** Logistic regressions between food insecurity and diabetes risk factors in a lower-income (300 % federal poverty line and below) sample of adolescents aged 12–19 years (*n* 3412) in NHANES cycles 2007–2016*

		Model 1†				Model 2‡			
	*n*	OR	95 % CI LL	95 % CI UL	*P*	OR	95 % CI LL	95 % CI UL	*P*
**HbA1C (%)** §	3412								
Full food security||		Ref.				Ref.			
Marginal food security		1·37	0·91	2·08	0·67	1·13	0·74	1·70	0·83
Food insecurity		1·61	1·10	2·36	0·06	1·37	0·95	1·98	0·12
* P*-for-trend					**0·01** ¶				0·09
**Fasting plasma glucose (mg/dL)**	1507								
Full food security		Ref.				Ref.			
Marginal food security		1·01	0·58	1·77	0·94	1·01	0·58	1·76	0·93
Food insecurity		0·98	0·69	1·39	0·89	0·98	0·70	1·38	0·90
*P*-for-trend					0·92				0·93
**HOMA-IR**	1457								
Full food security		Ref.				Ref.			
Marginal food security		1·16	0·73	1·85	0·72	0·96	0·60	1·54	0·90
Food insecurity		1·16	0·85	1·60	0·58	0·98	0·70	1·37	0·97
* P*-for-trend					0·34				0·89
**OGTT (mg/dL)**	1323								
Full food security		Ref.				Ref.			
Marginal food security		0·75	0·33	1·72	0·31	0·64	0·27	1·49	0·18
Food insecurity		1·26	0·68	2·34	0·23	1·21	0·69	2·15	0·16
* P*-for-trend					0·51				0·56

NHANES, National Health and Nutrition Examination Survey; LL, lower limit; UL, upper limit; mg/dL, milligram/decilitre; HOMA-IR, homoeostatic model assessment – insulin resistance; OGTT, oral glucose tolerance test.

* All analyses were conducted using survey procedures that account for the complex survey design including the strata, clusters and weights.

† Model 1 is age- and sex-adjusted.

‡ Model 2 is adjusted for adolescent age, sex, race/ethnicity, vigorous recreational activity, moderate recreational activity, sedentary time; household respondent education, marital status and income (linear and quadratic term).

§ The cut-points for HbA1C, fasting plasma glucose and OGTT are based on clinical cut-points. The value of the cut-point for HOMA-IR is the 75th percentile of that biomarker in the broader adolescent population (i.e. all adolescents aged 12–19 years who had data on the biomarker and fasted for 9–24 h).

|| According to the United States Department of Agriculture (USDA), full food security is defined as ‘no reported indications of food-access problems or limitations’. Marginal food security is defined as ‘one or two reported indications—typically of anxiety over food sufficiency or shortage of food in the house. Little or no indication of changes in diets or food intake’. Food insecurity is defined as ‘a household-level economic and social condition of limited or uncertain access to adequate food’. These definitions are from the USDA Economic Research Service (ERS): https://www.ers.usda.gov/topics/food-nutrition-assistance/food-security-in-the-us/definitions-of-food-security.aspx.

¶ Statistically significant estimates at alpha = 0·05 are bolded.

## Discussion

In this large national sample, we found that food insecurity was associated with slightly higher mean HbA1C after adjustment for household and adolescent characteristics. However, the effect size is small, and no associations were found with other diabetes risk factors. Moreover, it is important to consider that Fulay *et al.* (2021)^(22)^ found no associations between food insecurity and CVD risk factors in a similar national sample of lower-income US adolescents. This research suggests a slight association between food insecurity and diabetes risk among lower-income US adolescents. Future research could determine if this disparity is clinically meaningful and/or if the association may become more clinically relevant over time.

Our finding is consistent with the findings of two previous studies^(19,20)^. The first study by Lee *et al.* found an association between food insecurity and 1·94 higher odds of prediabetes via HbA1C in a national sample of US adolescents aged 12–19 years from NHANES cycles 2003–2014^(20)^. Similar to our analysis, they used the HbA1C cut-point of 5·7 %, but their classification of food insecurity differed such that they considered marginal food security, low food security and very low food security to be ‘food insecure^(20)^’. Our study separated the marginal food security from the other more severe food insecurity groups, per USDA guidelines, and also examined whether there was a gradient in the association between severity of food insecurity and diabetes risk. The second study, by Duke, found that food insecurity (assessed via a single question on skipping meals) was associated with 1·92 higher odds of self-reported prediabetes in a sample of adolescents aged 12–19 years residing in Minnesota, USA^(19)^. The differences in food insecurity categorisation/measurement in these two studies compared with ours likely explain why our findings are smaller in magnitude, although overall consistent. Furthermore, this study aligns with the research of Malik *et al.* who used data from the SEARCH for Diabetes in Youth study to find that US individuals aged 10–35 years with type 2 diabetes, as determined via a health professional diagnosis, were more likely to come from households experiencing food insecurity (i.e. low and very low food security)^(31)^. On the other hand, Marjerrison *et al.* found that there was an initial association between food insecurity and higher HbA1C in Canadian youth, aged on average ∼12 years, that attenuated after multivariable adjustment^(32)^. However, it is important to note that Canadian societal factors might differ from those in the USA and thus, this finding may not apply to US populations.

Although food insecurity was associated with HbA1C in this study, it is important to note that there were no associations found between food insecurity and FPG, HOMA-IR and OGTT levels. This may be because HbA1c represents a 3 month average of glycaemia and has much less within-person variability with repeated measurements compared with glucose or insulin measures^(33,34)^. However, the differential results between HbA1C and the other biomarkers still merit further investigation, particularly in light of the small HbA1C effect size.

It is important to consider nutritional and behavioural factors that might be driving the association between food insecurity and slightly higher HbA1C in lower-income US adolescents. Food insecurity has been associated with poorer dietary quality^(16)^ and diabetes risk^(13)^ in US adults. In US children, Landry *et al.* ^(35)^ and Fram *et al.* ^(36)^ found that food insecurity was associated with higher intake of sugars. Therefore, it is possible that specific components of diet related to diabetes risk are being overconsumed in populations with food insecurity. However, a systematic review by Hanson and Connor suggests that the association between food insecurity and dietary quality in US children (including adolescents) is mixed^(16)^. It is important to consider that dietary quality in US adolescents overall is poor^(37)^ and current dietary assessment methods may not consistently detect small differences in intake. In general, there is some evidence that diet may play role in this association between food insecurity and diabetes risk factors. A small association between food insecurity and diet may explain our smaller effect size for HbA1C and null findings for the other diabetes risk factors.

Furthermore, food insecurity has been associated with loss-of-control eating in adolescents^(38)^ and binge-eating in children with obesity^(39)^. It is possible, therefore, that eating behaviour differences could potentially explain the association between food insecurity and higher HbA1C. It has been proven that both voluntary and involuntary food restriction can cause changes in eating behaviour^(40)^. Binge and loss-of-control eating patterns have been linked to higher insulin resistance, in response to higher calorie/fat intake^(41)^, and higher type 2 diabetes risk^(42,43)^. It is possible that if youth with food insecurity are more likely to consume excessively large portions when food is available, this pattern could impact type 2 diabetes risk factors. Given the irregularity of such patterns, HbA1C might be more likely to capture the long-term impact of such behaviour compared with one-time fasting measures.

Further investigation of adolescent eating behaviour in response to food insecurity might elucidate potential mechanisms for future diabetes risk.

The public health implications of a potential association between food insecurity and diabetes risk among US adolescents could be important. It is possible that food insecurity could play a small contributing role in the rising incidence of type 2 diabetes^(7)^ among adolescents, a trend associated with higher disease burden^(10)^. Although the mechanism for the association between food insecurity and higher HbA1C in this age group is unclear, interventions that improve food security could help ameliorate this trend as well as provide numerous other health benefits. In particular, improvements to current federal nutrition assistance programmes may help reduce food insecurity in adolescents. For example, the Supplemental Nutrition Assistance Program (SNAP) provides benefits for low-income US households to purchase foods^(44)^. Adolescents, their parents and policy advocates believe that this programme assists in alleviating adolescent food insecurity and improvements such as higher benefits and less restrictive eligibility could further improve food insecurity in this age group^(45)^. Additionally, healthy incentive programmes such as Double-Up Food Bucks, a programme associated with SNAP that encourages fruit and vegetable purchases using a 2-for-1 system, could improve both food security and dietary quality^(46)^ which could help reduce type 2 diabetes risk. Meanwhile, universal school meals (which enable free school meals for all students) could help alleviate food insecurity^(47)^ and thus improve type 2 diabetes risk. The spillover effects of such programmes would likely also benefit other aspects of mental and physical health for this age group.

Strengths of this study include the systematic measurement of food insecurity in a lower-income population, the evaluation of multiple measures of glycaemia, including all of the formal tests used for assessing diabetes risk as well as insulin resistance measures, its large study sample and the nationally representative sample of lower-income US adolescents (which included an oversampling of non-Hispanic Black and Hispanic populations). Limitations include the cross-sectional design, which precludes causation and possible residual confounding due to unmeasured variables. Social desirability bias might exist for sensitive variables such as food insecurity and income while recall bias may exist for fasting status and time reporting^(48)^. While it is impossible to completely correct for such biases, NHANES has strict protocols including interviewer training and data cleaning to maximise the collection of highly accurate data^(21)^. Another potential limitation is that this analysis included data collected over the span of 10 years, and the association between food insecurity and diabetes risk factors may vary from year-to-year. Finally, household food insecurity, as reported by the parent/caregiver in the household, may not capture the experience of food insecurity for the adolescent. Studies that have used child-reported food insecurity have found differential associations with children’s health outcomes when compared with parent/caregiver reports^(49)^. That being said, there is currently no consistent approach for adolescent reported food insecurity in the USA, which represents an important goal of future research.

In summary, household food insecurity was associated with slightly higher mean HbA1C levels in a national sample, but not with short-term glucose levels, insulin resistance or OGTT in a lower-income sample of US adolescents. Thus, there is some evidence to suggest a slight association between food insecurity and diabetes risk in lower-income US adolescents, which has been corroborated by a few other studies^(19,20)^. Additional research is needed to confirm these findings and examine a potential causal mechanism for this association, such as investigating the role of dietary quality and eating behaviour. Longitudinal studies are needed to examine the potential clinical relevance of these findings over time. In particular, future research should examine the longitudinal association between repeated measures of food insecurity and diabetes risk factors over the lifespan to see how the association between food insecurity and diabetes might change as an individual with continual food insecurity transitions from adolescence to adulthood. Policies and programmes that increase access to nutritious foods could disproportionately benefit US adolescents with food insecurity in terms of their dietary intake, diabetes risk and overall health^(50)^.
